# Logistic Model of Phase Transformation of Hardening Concrete

**DOI:** 10.3390/ma15134403

**Published:** 2022-06-22

**Authors:** Jan Ślusarek, Artur Nowoświat, Marcelina Olechowska

**Affiliations:** Faculty of Civil Engineering, Silesian University of Technology, Akademicka 2A, 44-100 Gliwice, Poland; jan.slusarek@polsl.pl (J.Ś.); marcelina.olechowska@polsl.pl (M.O.)

**Keywords:** cement paste, superplasticizer, viscous liquid, autocatalytic transformation, logistic trend

## Abstract

The objective of this study was to analyze the effects of the modification of cementitious materials with the admixture of a superplasticizer and mineral additive in the form of microsilica. We analyzed the hardening process of cementitious materials as an autocatalytic transformation from a viscous liquid to a pseudo-solid body. The main achievement of the research involved the identification of changes in the content of the solid phase during the hardening of concrete mix as a model of the logistic curve. The phase transformation process begins with a viscous liquid which consists of water, cement, microsilica, superplasticizer and sand. The laboratory tests comprised research on the development of the kinetics of hardening heat of binder cement pastes and the development of mechanical properties of concrete in the hardening process. Three groups of mixtures with different concentrations of binders, determined by different water–binder indexes, were used. The first group was made up by cement paste consisting of cement and water. The second group contained cement paste from the first group supplemented with a superplasticizer, and the third group comprised the cement paste as in the second group, but additionally modified with microsilica. Using appropriate analyses, we defined a mathematical model of the phase transformation process. The model was then used for computer-aided numerical analyses. This made it possible to compare the relevant parameters of the logistic curve obtained for the seven different concrete mixes analyzed. Active impact of the applied mineral additive (microsilica) and that of chemical admixture in the form of a superplasticizer was demonstrated. All approximations of the measurement results with the use of a logistic curve had a determination level of over 0.98, indicating high agreement.

## 1. Introduction

The properties of hardening cement have long constituted an interesting subject of studies for researchers worldwide. The relevant studies address the homogenization process of hardening cement pastes [1]. The complexity of hardening processes, which evolve over time [2], is attributed to a strong impact of environmental factors, which include temperature or humidity [3]. Research dedicated to solving such complex problems has been carried out by Li et al. [4] and Mallick et al. [5]. Factors that adversely affect hydratation have also been considered. Among other things, the use of natural material fibers can have a negative impact, as they result in low mechanical properties and poor adhesion on the matrix [6]. Another issue involves the behavior of lime-containing composite cements hydrated at different water-to-binder ratios. Studies such as that carried out by Zając et al. [7] provide a better understanding of the impact of the added supplementary cementitious materials on the performance of composite cement. From the viewpoint of hydratation and the resulting set of impact phases of the added cementitious materials, the problem is now better recognized [8,9]. Very reactive Portland cement clinker powder generates a series of reactions when mixed with water—the so-called hydratation process, comprising crystal phases, surface reactions, gel formation and precipitation of new phases [10].

Thermal effects concerning the hydratation of cements are of particular importance in massive concrete structures [11]. The difference in temperature between the inside and the relatively quickly cooled outside surface of a concrete element, which is caused by heat released in the cement hydratation process, leads to the development of thermal stresses. Under extreme conditions, it may result in cracking of the entire volume of a concrete element, which shortens its durability and lifetime [12]. A thorough understanding of the hydratation characteristics of cement paste backfill (CPB), as an analysis of the causes of low strength, large deformations and high costs in the early-age hardening period, was investigated by Xin et al. [13]. The early-age hydratation mechanism was investigated by Zhao [14]. They also analyzed the hydratation characteristics and kinetic parameters of a cement mix having a different admixture substitution rate and temperature. Many researchers have studied the effect of admixtures on cement hydratation. For example, chemical admixtures such as Na_2_CO_3_, Na_2_HCO_3_ or Ca (HCO_3_) can improve early-age hydratation of cement [15,16]. The experimental results by Li et al. [17] also demonstrated that the hydratation period of PC paste was improved by increasing the w/c ratio due to the improvement of space available for the hydratation product growth. The early-age hydratation of cement largely determined the setting time and early-age strength of concrete [18]. Generally, it can be observed that in order to improve the workability of Portland cement, many admixtures are used in the cement industry to optimize the hydratation process and to improve the mechanical properties [19,20,21,22]. On the other hand, Zhang et al. [23] demonstrated that acidic chemical additives can inhibit the hydrolyzing–bridging reaction of Mg^2+^ ions in the liquid phase and delay the hydratation reaction. In Ref. [24], it was demonstrated that after adding hydrate seed–polycarboxylate (C-S-Hs-PCE) and sodium sulfate (SS), the setting time was shortened and the strength of cement paste improved drastically. The time of cement hydratation is also influenced by nanoparticles [25,26], or by mineral admixtures through the impact of backfill [27,28].

In this paper, we attempt to describe the kinetics of phase transformation of the hardening concrete using the developed mathematical model. A logistic model was used and parameters were estimated using the least squares method with the application of empirical results. It turned out that the content of the solid phase as a function of concrete hardening time changes in line with the logistic function, and the correlation between the experimental results and the approximation with this function is not lower than 0.98. This means that the correlation coefficient is at the level of 0.99. These results mean that in each case described in this paper, the logistic function describes at least 98% of the results of the experiment. Moreover, the applied superplasticizer admixtures and mineral additives in the form of microsilica changed the reaction rate. The analysis involved the hardening process of cement materials as an autocatalytic transformation from a viscous liquid to a “pseudo-solid” body.

## 2. Materials and Methods

### 2.1. Characteristics of the Applied Materials

To analyze the hardening process of cementitious materials, some parameters described in the works of the co-author of this article were applied [29,30,31]. We attempted to analyze the effects of the modification of cementitious materials with the admixture of superplasticizer and mineral additive in the form of microsilica. The components used to subject the mixtures to analysis include bridge cement, silica fume, superplasticizer, washed sand and basalt grit. Table 1 presents the parameters of the investigated mixes. The PC mix (plain concrete) was developed from bridge cement 45-Rejowiec. The mixes SP-1, SP-2 and SP-3 contain cement 45-Rejowiec and superplasticizer Melment L10/40% in the amount of 1.25% of the cement mix mass. The mixes SF-4, SF-5 and SF-6 contain cement 45-Rejowiec, the admixture of amorphous silica in the amount of 10% of the total mass of the binder, and the superplasticizer Melment L 10/40% in the amount of 2.5% of the binder mass.

The graining curve, encompassing a mixture of washed sand 0–2 and basalt grit 4–8, was selected using the experimental method, yielding the maximum tightness at the level of 0.729. The Melment superplasticizer we used was produced based on water-soluble sulfonated polycondensation products of melanin and formaldehyde. The sulfonation was effected by introducing a sulfone group, SO_3_H, into the molecule of the organic compound. The Melment L 10/40% used in the tests was a 40% aqueous solution of the active substance. The density of water solution was 1.258 g/cm^3^, while that of dry mass was 2.05 g/cm^3^.

**Figure 1:**
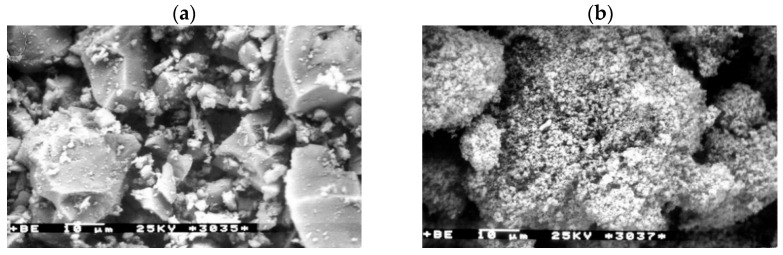
(**a**) View of the microstructure of bridge cement 45 with the specific surface area of 3011 cm^2^/g. (**b**) View of the microstructure of microsilica with the specific surface area of 180,000 cm^2^/g.

Figure 1a,b show the photos of the microstructure of the used cement and microsilica. The specific surface areas of these materials differ significantly. For cement, the surface area is 3011 cm^2^/g, and for microsilica, it is as large as 180,000 cm^2^/g.

### 2.2. Investigation Methods of the Hardening Kinetics of Cementitious Materials

For the research on hardening kinetics of cementitious materials, samples with the dimensions of 4 × 4 × 16 cm were used. The selection of small samples was enforced by limiting the maximum size of basalt grit grains used for the preparation of individual concretes to 8 mm. All samples were protected with PVC foil to prevent moisture exchange with the environment. Samples stored at 298 K were used to analyze the hardening kinetics of cementitious materials.

### 2.3. Mathematical Model

**Figure 2:**
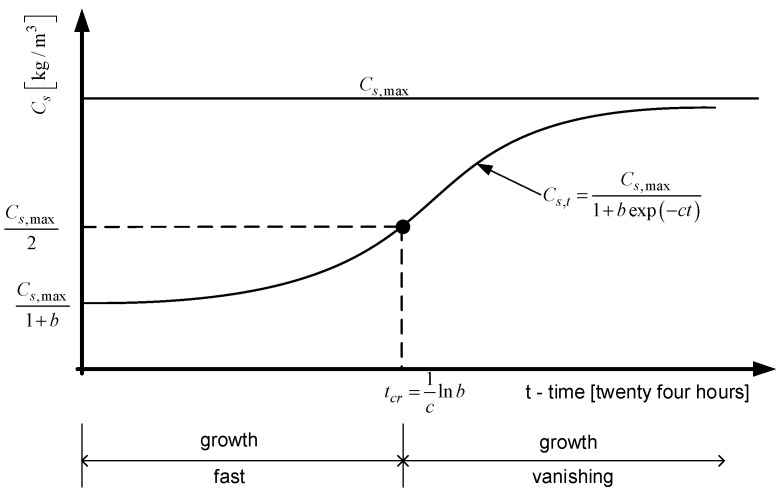
Graph of the logistic curve of the Equation (1).

The logistic curve of the following equation was adopted as a model for the solid phase content of the hardening material after time *t*:

(1) $$C_{s,t}=\frac{C_{s,\max}}{1+b\exp\left(-ct\right)},$$

where *C_s_*_,*t*_ is the content of solid phase of the hardening material after time *t*; *C_s_*_,*max*_, *b* and *c* are parameters of the logistic curve; and *t* is the duration of phase transformation (24 h). The model is presented graphically in Figure 2.

Logistic models for the described process were used for the first time by Ślusarek [30]. This theory has also been applied in this paper.

Based on the logistic curve presented in Figure 2, several characteristic points can be distinguished. The initial content of solid phase at time *t* = 0 meets the condition:

(2) $$C_{s,0}=\frac{C_{s,\max}}{1+b},$$

The content of solid phase after the critical time *t_cr_* is $\frac{C_{s,\max}}{2}$, where the critical time is:

(3) $$t_{cr}=\frac{1}{C}\ln b,$$

In order to estimate the function approximating the measurement results of *C_s_*, the non-linear least squares method was applied with the use of the Gauss–Newton method. This method involves a sequence of successive applications of the least squares method with the defined observation matrices of explanatory variables and with the observation vector of the dependent variable. The general form of the function under consideration can be written as $Y_{i}=\frac{a}{1+be^{-cx_{i}}}$.

### 2.4. Validation of the Model

There are many validation methods of mathematical or numerical models. Here, we can refer to the Stern method [32] applied, e.g., for acoustic models [33]. However, in this case, in addition to the determined confidence level, Schlesinger’s approach [34] was used, which consists of checking whether the model has in its field of application a sufficient level of validity. For this purpose, variance analysis was used. It involves testing hypotheses which state that the mean value obtained from the characteristic of time series from the simulation is equal to the mean value of the time series obtained from the observation of empirical results. The validation was carried out for the plain concrete PC. The model parameters and the model’s validation were realized using the STATISTICA software.

### 2.5. Concrete PC

**Figure 3:**
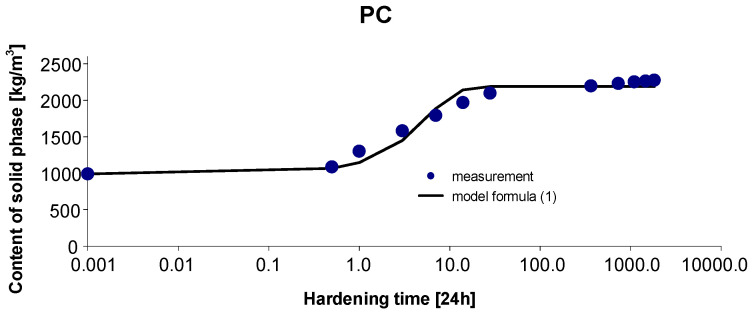
Measurement results and the approximating function of phase transformation kinetics for PC concrete.

Based on the measurement results presented in Figure 3, the parameters of the model of the function approximating these results, described by the Equation (1), were estimated. The determined 95% confidence interval of the estimated parameters is as follows: $C_{s,\max}\in \langle 2111.717;\;2296.489\rangle$, b∈〈0.752; 1.277〉, c∈〈0.095; 0.344〉. By estimating the parameters of Model (1) with the Gauss–Newton method, we obtain $C_{s,\max}=2207.872$, b=1.0146 and c=0.2775. The standard errors of the estimate are $\delta_{C_{s,\max}}=43.840$, $\delta_{b}=0.1160$ and $\delta_{c}=0.0551$, respectively. The test probabilities for each estimated parameter were p<<0.05, indicating the statistical significance of the results. In addition, the hypothesis on the normal distribution of residuals was verified by the Shapiro–Wilk test. The test statistic with the test probability *p* = 0.79113 is SW-W = 0.96051. Since the calculated value of the test probability *p* is higher than the adopted significance level of the test of 0.05, we conclude that there is no reason to reject the hypothesis H0, which assumes a normal distribution of residuals. Ultimately, Model (1) has the following form:

(4) $$C_{s,t}=\frac{2207.872}{1+1.0146\exp\left(-0.2775\;t\right)},$$

The graph of the approximating Function (4) with the measurement points of the content of solid phase as a function of hardening time is presented in Figure 3.

The coefficient of determination is $r^{2}=0.989$.

Basic validation based on the assessment of the confidence level showed that because p<<0.05, the model correctly approximates the empirical results. Additionally, standard errors of model estimation were determined.

The additional validation was initiated by checking the homogeneity of variance. For this purpose, we assumed the following:

**H0**: *Homogeneity of variance is present.*

**H1**: *Homogeneity of variance is absent.*

The hypothesis was verified using the F test, for which we obtained p=0.8477. Since the obtained *p*-value is higher than 0.05, there is no reason to reject the null hypothesis of homogeneity of variance. In the next step, the hypothesis of the equality of means is checked.

**H0**: *The means of the time series of empirical results do not differ significantly from the means obtained from the model.*

**H1**: *The hypothesis opposite to H0.*

The verification of the hypotheses was performed using the t-test for independent samples, assuming the homogeneity of variance, which has already been verified. Since the test value p=0.9617 is higher than 0.05, there is no reason to reject the null hypothesis. Thus, the mean value of the empirical results does not differ significantly from the mean value of the model. To be precise, these values are ${\bar{x}}_{measur.}=1836\;\mathrm{kg}/m^{3}$, ${\bar{x}}_{sym.}=1826\;\mathrm{kg}/m^{3}$.

In sum, it can be stated that the validation was positive and the proposed logistic model effectively approximates the empirical results.

## 3. Results and Discussion

When analyzing the kinetics of hardening concrete, a certain tendency can be observed. At the beginning of the process, a fast growth of the analyzed parameter is observed (e.g., compressive strength), followed by its slow (vanishing) growth. The initial parameters of the analyzed cementitious materials are presented in Table 2.

The quantity *C_L_*_,0_ represents a viscous liquid at the beginning of the phase transformation process. The quantity *C_s_*_,0_ stands for the content of the solid phase at the beginning of the phase transformation process. It was assumed for the analysis that the solid phase at the beginning of the process was only made up by coarse aggregate.

The phase transformation mechanism can be described by the equations:

(5) $$C_{L}\left(t\right)=C_{L,0}-\alpha \left(t\right)\cdot C_{L,0},$$

(6) $$C_{s}\left(t\right)=C_{s,0}+\alpha \left(t\right)\cdot C_{L,0},$$

where *α*(*t*) is the value of the phase transformation degree (hardening degree of the cementitious material) at the observation time *t*. In that case, the observation time was 1825 days. The degree of structural transformations is determined from the formula [29]:

(7) $$\alpha =\frac{R_{c}}{R_{\max}},$$

where *R* is the compressive strength of concrete at a given point in the development stage of the structure and *R*_max_ is the concrete strength calculated for *x = x*_max_ determined from the Formula (8) [29]:

(8) $$R_{c}=R_{c,0}\cdot x^{a}\cdot \exp\left[b\left(1-x\right)\right],$$

where *R_c_*_,0_ is the theoretical compressive strength of concrete for *x* = 1 (MPa), *x* is the porosity coefficient of concrete structure and *a* and *b* are kinetic parameters, determined by the multiple regression method, dependent on the type of concrete structure.

The porosity coefficient of concrete structure is determined using the formula [29]:

(9) $$x=\frac{\omega_{g}}{\omega_{g}+\omega_{c}+\omega_{a}},$$

where *ω_g_, ω_c_* and *ω_a_* stand for the volume of gel, capillary and air pores, respectively, referenced to the mass unit of the binder (dm^3^/kg).

Thus, taking into account the appropriate values of the volume of pores, we obtain [29]:

(10) $$x=\frac{0.28\alpha \left(\frac{1}{\rho_{s}}+\omega_{H}+\omega_{p}-V_{s}\right)}{0.28\alpha \left(\frac{1}{\rho_{s}}+\omega_{H}+\omega_{p}-V_{s}\right)+\omega -\left(\omega_{H}+\omega_{p}\right)\cdot \alpha +\omega_{a}},$$

where *ω* is the water/binder ratio (*ω* = w/s) (dm^3^/kg), *w* is the initial water content in concrete (dm^3^/m^3^), s is the binder mass in concrete (kg/m^3^), *ω_H_* is the volume of chemically bonded water by the mass unit of the binder (dm^3^/kg), *ω_p_* is the volume of the extraneous water remaining in the structure of the binder gel referenced to the mass unit of the binder (dm^3^/kg), *α* is the (conversion) degree of structural transformation of the cementitious material, *ρ_s_* is the binder density and *V_s_* is the volume change in the water–binder system referenced to the mass unit of the binder (contraction).

Table 3 presents the maximum values of the described structures.

The obtained values of the degrees of structural transformations (conversion degrees) of individual cementitious materials are presented in Table 4.

Using the nonlinear least squares method described in Section 2.3, with the application of the Gauss–Newton method, the parameters of the model were estimated based on the measurements (1).

### 3.1. Materials SP-1

As before, the 95% confidence interval of the estimated parameters was determined: $C_{s,\max}\in \langle 1988.798;\;2194.730\rangle$, b∈〈0.684; 1.134〉, c∈〈0.037; 0.149〉. By estimating the parameters of Model (1) using the Gauss–Newton method, we obtain $C_{s,\max}=2104.021$, b=1.1301 and c=0.1215. The standard errors of the estimate are $\delta_{C_{s,\max}}=45.517$, $\delta_{b}=0.0995$ and $\delta_{c}=0.0247$, respectively. The test probabilities for each estimated parameter were p<<0.05, which proves the statistical significance of the results. In addition, the hypothesis on the normal distribution of residuals was verified by the Shapiro–Wilk test. The test statistic with the test probability of *p* = 0.33195 is SW-W = 0.9252. Since the calculated value of the test probability *p* is greater than the adopted significance level of the test of 0.05, we conclude that there is no reason to reject the hypothesis H0, which assumes a normal distribution of residuals. Ultimately, Model (1) has the form:

(11) $$C_{s,t}=\frac{2104.021}{1+1.1301\exp\left(-0.1215\;t\right)},$$

**Figure 4:**
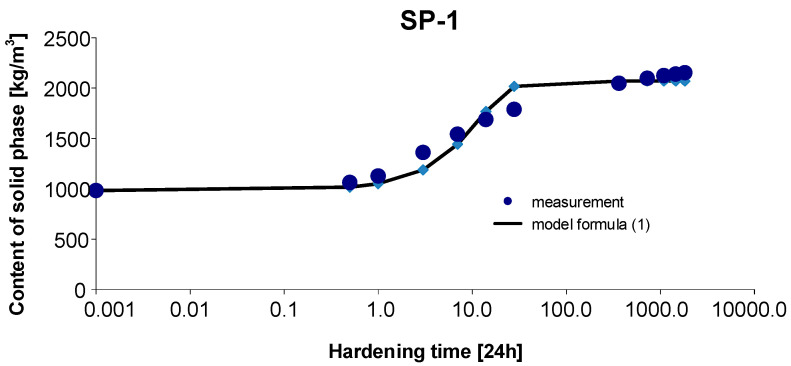
Measurement results and the approximating function of phase transformation kinetics for concrete SP-1.

The graph of the approximating Function (11) with the measurement points of the content of solid phase as a function of hardening time is presented in Figure 4.

The coefficient of determination is $r^{2}=0.986$.

### 3.2. Materials SP-2

As before, the 95% confidence interval of the estimated parameters was determined: $C_{s,\max}\in \langle 1959.923;\;2155.653\rangle$, b∈〈0.645; 1.056〉, c∈〈0.037; 0.145〉. By estimating the parameters of Model (1) using the Gauss–Newton method, we obtain $C_{s,\max}=2072.285$, b=1.0828 and c=0.1221. The standard errors of the estimate are $\delta_{C_{s,\max}}=43.262$, $\delta_{b}=0.0909$ and $\delta_{c}=0.0238$, respectively. The test probabilities for each estimated parameter were p<<0.05, which proves the statistical significance of the results. In addition, the hypothesis on the normal distribution of residuals was verified by means of the Shapiro–Wilk test. The test statistic with the test probability of *p* = 0.33532 is SW-W = 0.9256. Since the calculated value of the test probability *p* is greater than the adopted significance level of the test of 0.05, we conclude that there is no reason to reject the hypothesis H0, which assumes a normal distribution of residuals. Ultimately, Model (1) has the form:

(12) $$C_{s,t}=\frac{2072.285}{1+1.10828\exp\left(-0.1221\;t\right)},$$

**Figure 5:**
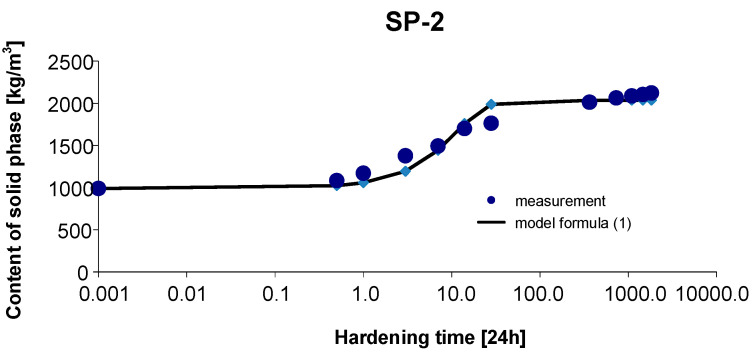
Measurement results and the approximating function of phase transformation kinetics for concrete SP-2.

The graph of the approximating Function (12) with the measurement points of the content of solid phase as a function of hardening time is presented in Figure 5.

The coefficient of determination is $r^{2}=0.986$.

### 3.3. Materials SP-3

As before, the 95% confidence interval of the estimated parameters was determined: $C_{s,\max}\in \langle 2006.412;\;2204.614\rangle$, b∈〈0.682; 1.114〉, c∈〈0.038; 0.126〉. By estimating the parameters of Model (1) using the Gauss–Newton method, we obtain $C_{s,\max}=2125.437$, b=1.1376 and c=0.1071. The standard errors of the estimate are $\delta_{C_{s,\max}}=43.808$, $\delta_{b}=0.0926$ and $\delta_{c}=0.0196$, respectively. The test probabilities for each estimated parameter were p<<0.05, which proves the statistical significance of the results. In addition, the hypothesis on the normal distribution of residuals was verified by means of the Shapiro–Wilk test. The test statistic with the test probability *p* = 0.84246 is SW-W = 0.96426. Since the calculated value of the test probability *p* is greater than the assumed significance level of the test of 0.05, we conclude that there is no reason to reject the hypothesis H0, which assumes a normal distribution of residuals. Ultimately, Model (1) has the form:

(13) $$C_{s,t}=\frac{2125.437}{1+1.1376\exp\left(-0.1071\;t\right)},$$

**Figure 6:**
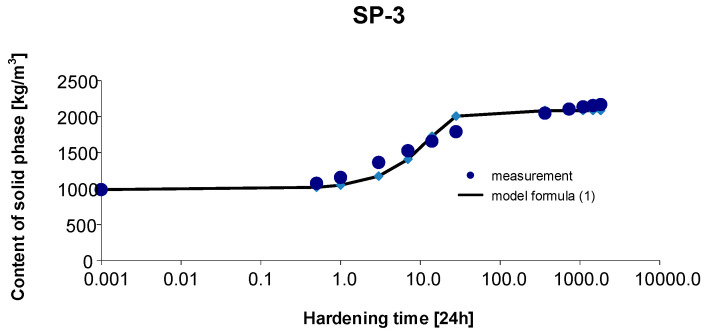
Measurement results and the approximating function of phase transformation kinetics for concrete SP-3.

The graph of the approximating Function (13) with the measurement points of the content of solid phase as a function of hardening time is presented in Figure 6.

The coefficient of determination is $r^{2}=0.985$.

### 3.4. Materials SF-4

As before, the 95% confidence interval of the estimated parameters was determined: $C_{s,\max}\in \langle 2287.309;\;2446.571\rangle$, b∈〈0.944; 1.379〉, c∈〈0.101; 0.218〉. By estimating the parameters of Model (1) using the Gauss–Newton method, we obtain $C_{s,\max}=2366.94$, b=1.1619 and c=0.1595. The standard errors of the estimate are $\delta_{C_{s,\max}}=36.201$, $\delta_{b}=0.0962$ and $\delta_{c}=0.0258$, respectively. The test probabilities for each estimated parameter were p<<0.05, which proves the statistical significance of the results. In addition, the hypothesis on the normal distribution of residuals was verified by means of the Shapiro–Wilk test. The test statistic with the test probability *p* = 0.6501 is SW-W = 0.9509. Since the calculated value of the test probability *p* is greater than the adopted significance level of the test of 0.05, we conclude that there is no reason to reject the hypothesis H0, which assumes a normal distribution of residuals. Ultimately, Model (1) has the form:

(14) $$C_{s,t}=\frac{2366.94}{1+1.1619\exp\left(-0.1595\;t\right)},$$

**Figure 7:**
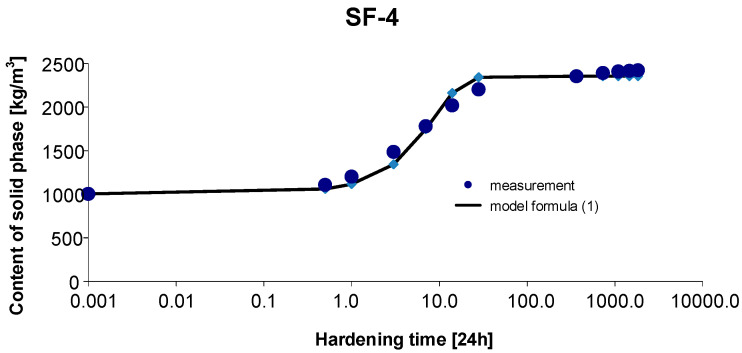
Measurement results and the approximating function of phase transformation kinetics for concrete SF-4.

The graph of the approximating Function (14) with the measurement points of the content of solid phase as a function of hardening time is presented in Figure 7.

The coefficient of determination is $r^{2}=0.995$.

### 3.5. Materials SF-5

As before, the 95% confidence interval of the estimated parameters was determined: $C_{s,\max}\in \langle 2282.620;\;2493.504\rangle$, b∈〈0.869; 1.473〉, c∈〈0.087; 0.283〉. By estimating the parameters of Model (1) using the Gauss–Newton method, we obtain $C_{s,\max}=2388.062$, b=1.1709 and c=0.1849. The standard errors of the estimate are $\delta_{C_{s,\max}}=46.611$, $\delta_{b}=0.1336$ and $\delta_{c}=0.0435$, respectively. The test probabilities for each estimated parameter were p<<0.05, which proves the statistical significance of the results. In addition, the hypothesis on the normal distribution of residuals was verified by means of the Shapiro–Wilk test. The test statistic with the test probability of *p* = 0.3173 is SW-W = 0.9236. Since the calculated value of the test probability *p* is greater than the assumed significance level of the test of 0.05, we conclude that there is no reason to reject the hypothesis H0, which assumes a normal distribution of residuals. Ultimately, Model (1) has the form:

(15) $$C_{s,t}=\frac{2388.062}{1+1.1709\exp\left(-0.1849\;t\right)}$$

**Figure 8:**
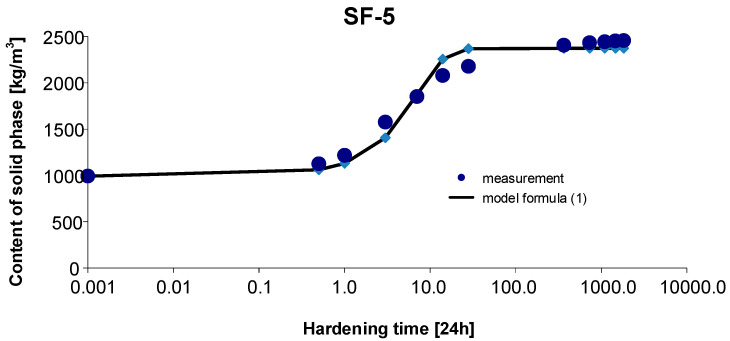
Measurement results and the approximating function of phase transformation kinetics for concrete SF-5.

The graph of the approximating Function (15) with the measurement points of the content of solid phase as a function of hardening time is presented in Figure 8.

The coefficient of determination is $r^{2}=0.992$.

### 3.6. Materials SF-6

As before, the 95% confidence interval of the estimated parameters was determined:, $C_{s,\max}\in \langle 2282.620;\;2493.504\rangle$, b∈〈0.869; 1.473〉, c∈〈0.087; 0.283〉. By estimating the parameters of Model (1) using the Gauss–Newton method, we obtain $C_{s,\max}=2388.062$, b=1.1709 and c=0.1849. The standard errors of the estimate are $\delta_{C_{s,\max}}=55.570$, $\delta_{b}=0.1126$ and $\delta_{c}=0.0201$, respectively. The test probabilities for each estimated parameter were p<<0.05, which proves the statistical significance of the results. In addition, the hypothesis on the normal distribution of residuals was verified by means of the Shapiro–Wilk test. The test statistic with the test probability *p* = 0.4626 is SW-W = 0.9372. Since the calculated value of the test probability *p* is greater than the adopted significance level of the test of 0.05, we conclude that there is no reason to reject the hypothesis H0, which assumes a normal distribution of residuals. Ultimately, Model (1) has the form:

(16) $$C_{s,t}=\frac{2447.645}{1+1.0\exp\left(-0.0842\;t\right)}$$

**Figure 9:**
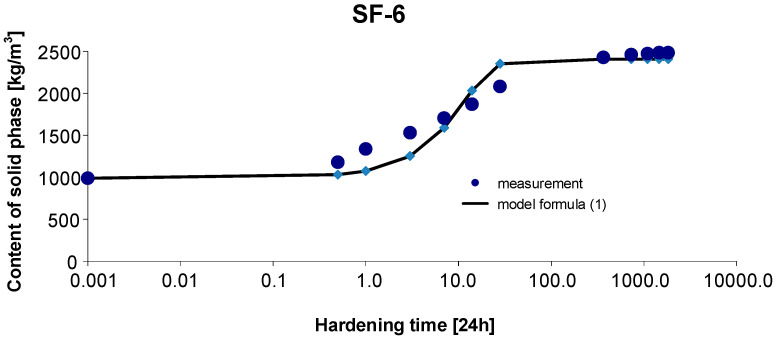
Measurement results and the approximating function of phase transformation kinetics for concrete SF-6.

The graph of the approximating Function (16) with the measurement points of the content of solid phase as a function of hardening time is presented in Figure 9.

The coefficient of determination is $r^{2}=0.990$.

Based on the determined models, we can determine the critical time *t_cr_* and initial content of the solid phase *C_s_*_,0_. The results are collected in Table 5.

It should be noted that the solid phase at the beginning of the process *C_s_*_,0_ is composed only of coarse aggregate. Moreover, the critical time *t_cr_* is the time in which the analyzed quantity *C_s_* (*t*) reaches the value of the half of *C_s_*_,max_.

**Figure 10:**
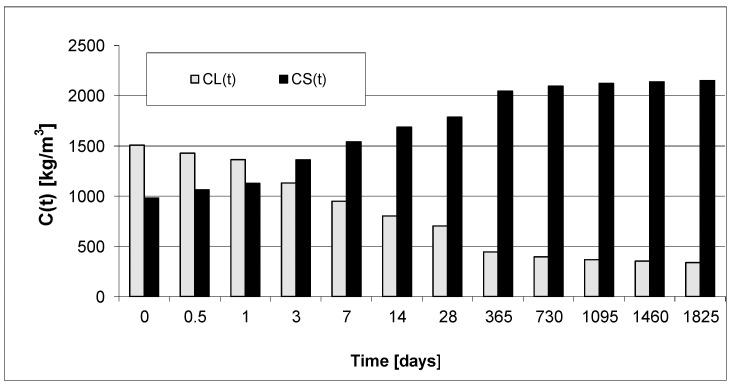
Concentration of liquid and solid phases (kg/m^3^) (Concrete PC).

**Figure 11:**
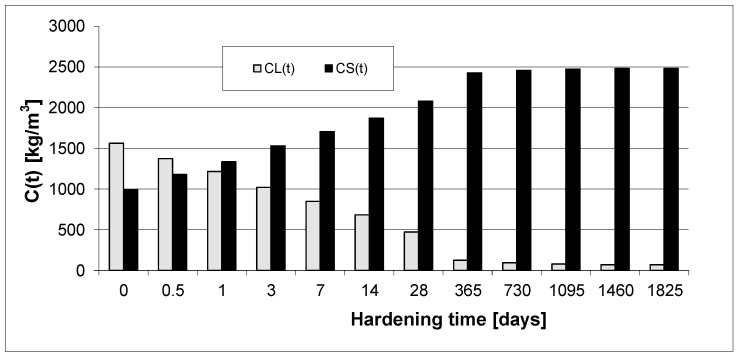
Concentration of liquid and solid phase (kg/m^3^) (Concrete SF-6).

In order to analyze the kinetics of phase transformations, Figure 10 and Figure 11 present exemplary diagrams of the transformations of the content of viscous liquid and solid phase over time for the concretes.

Here, we present the transformations in the mass of solid phase and viscous liquid only for plain concrete PC and for high-performance concrete (HPC) SF-6.

**Figure 12:**
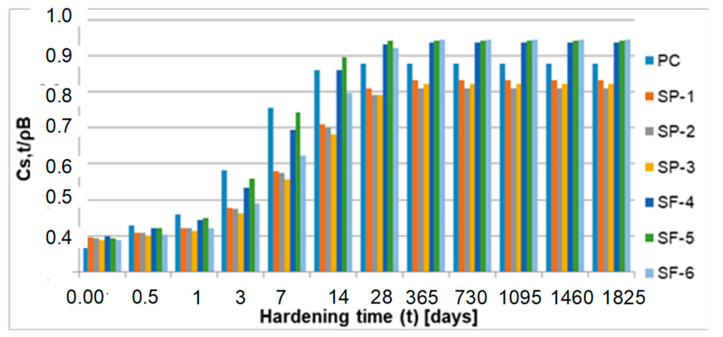
Mass of the solid phase over 5 years.

**Figure 13:**
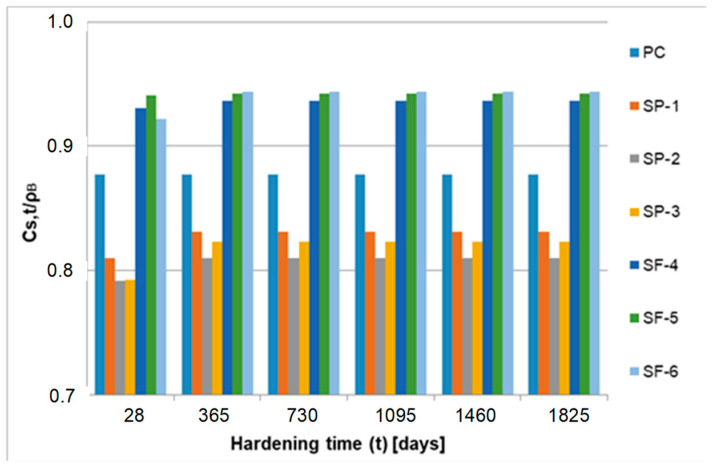
Mass of the solid phase over 14 days.

**Figure 14:**
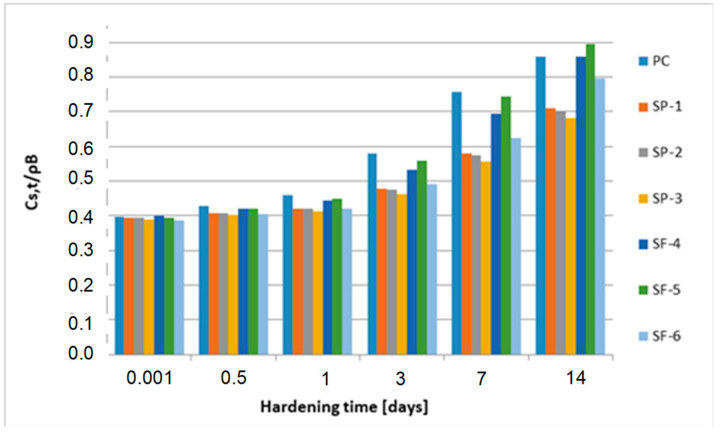
Mass of the solid phase over 28 ÷ 1825 days.

Figure 12, Figure 13 and Figure 14 show the graphs of solid phase mass over 28–1825 days for all analyzed concretes.

An important parameter of the logistic curve presented in Figure 1 is the quantity *C_s_*_,max_/2, which defines the mass of solid phase observed at the critical time *t_cr_*. For further analysis, the index described by Formula (17) was used:

(17) $$\frac{\Delta S\left(t_{cr}\right)}{t_{cr}}=\left[\frac{C_{s,\;\max}}{2}-C_{s,0}\right]/t_{cr}$$

**Figure 15:**
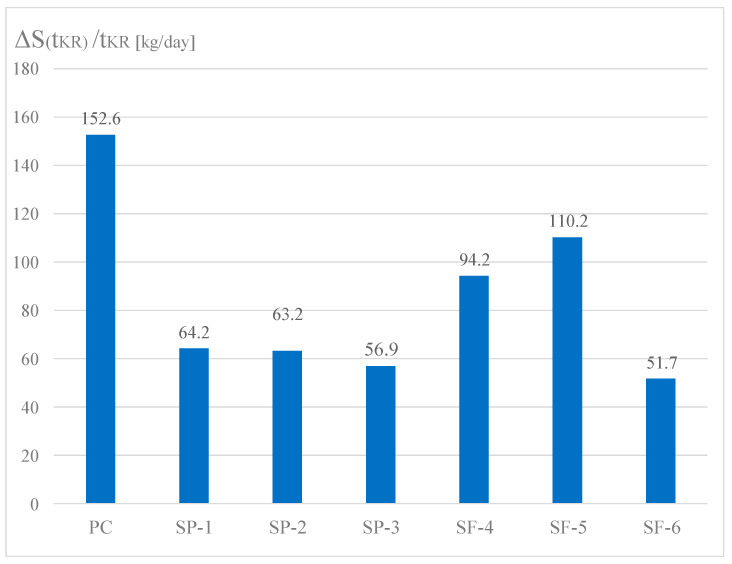
Increment in solid phase mass in the critical time for individual concretes.

The above parameter defines the rate of mass increment in the solid phase in the critical time (Figure 15). It turns out that during this time, the greatest increment in the solid phase is observed for plain concrete (PC), which does not contain chemical additives or natural admixtures.

Significantly less solid phase (only about 42%) is observed in concretes SP-1, SP-2 and SP-3 containing a superplasticizer, which appears to be effective in inhibiting the formation of solid phase in the initial time of transformation. In concretes SF-4, SF-5 and SF-6 containing both a superplasticizer and microsilica, a clear impact of microsilica is observed, which, due to its large specific surface, clearly weakens the inhibitory effect of the superplasticizer. The largest volume of solid phase mass during the critical time is gained in concrete SF-5, and the least in concrete SF-6. In concrete SF-4, a lower value by about 18% was observed, and in concrete SF-6, a value as much as about 37% lower was observed as compared to concrete SF-5. In subsequent analyses, we found that the highest value of the index *C_s_*_,*t*_/ρ_B_ was reported for PC concrete (without chemical admixtures and without mineral additives). It is clearly visible already after 3 days of hardening. After 28 days of hardening, this value stabilizes and practically does not change until the end of the observation (5 years). In the group of concretes SP-1 ÷ SP-3 (concretes containing a superplasticizer), more pronounced differences in the values of the index *C_s_*_,*t*_/ρ_B_ are observed only after 28 days. After 5 years, they reach the values of about 0.81 ÷ 0.83. In the group of concretes SF-4 ÷ SF-6 (concretes containing both a superplasticizer and microsilica), clear differences can be observed after 3 days of hardening. For concrete SF-6, an evidently higher value of the index *Cs,t*/ρ_B_ was observed over 7, 14 and 28 days. The values of this index stabilize after 1 year and they practically do not change until the end of the observation (5 years). After 5 years, the indexes reach the value of approximately 0.94 for all concretes from this group (concretes SF-4 ÷ SF-6). The analysis of the obtained test results indicates the active impact of the superplasticizer and microsilica on the kinetics of phase transformation processes, i.e., the transformation of cementitious material from a viscous liquid to a pseudo-solid body. The particles of superplasticizer are adsorbed on the binder grains and bring about their deflocculation by imparting an equal charge to their surfaces, which causes repulsive forces. Nevertheless, we observed that the superplasticizer blocks the progress of the phase transformation process due to its adsorption on the surfaces of binder grains. This is visible in the graphs presenting the relative mass increments of the solid phase, especially in Figure 12, i.e., in the first 14 days of the transformation process. The slowing and blocking effect of the superplasticizer is evident when comparing the values of *C_s_,_max_*/ρ_B_ obtained for concretes PC (w/s = 0.52) and 1 (w/s = 0.52). The value of this parameter for concrete PC is approximately 0.88, while for concrete 1 (containing a superplasticizer), it is only approximately 0.83. The pozzolanic microsilica reacts with portlandite Ca(OH)_2_, and hence, the size of Ca (OH)_2_ crystals and the degree of their orientation in relation to the aggregate grains are both decreasing, thereby strengthening this weak zone in the concrete. Microsilica with a large, developed surface easily reacts with Ca(OH)_2_, increasing the amount of hydrated calcium silicates of the CSH type (i.e., CaO-SiO_2_-H_2_O). The effect involving the impact of microsilica in the presence of a superplasticizer is revealed during the analysis of the parameter *C_s_*_,*t*_/ρ_B_, since the highest values of the *C_s_*_,*t*_/ρ_B_ index were observed in the group of concretes SF-4 ÷ SF-6. After 5 years of observation, the value of this index for these concretes is approximately 0.94.

## 4. Conclusions

A broader analysis of the kinetics of the hardening process of cementitious materials allows us to conclude that the rise in the values of the analyzed parameters over time is sufficiently described by the logistic curve of Model (1). In this article, we describe the phase transformation of hardening cementitious materials from a viscous liquid to a pseudo-solid body. The analyses allowed us to identify characteristic trends in the hardening process of various cementitious materials. The analysis involved plain concrete (PC concrete) with the water–binder ratio of 0.52; concretes modified with a superplasticizer with the water–binder ratios of 0.52, 0.47 and 0.42 (concretes SP-1, SP-2 and SP-3); and concretes modified with a superplasticizer and microsilica with the water–binder ratios of 0.42, 0.37 and 0.32 (concretes SF-4, SF-5 and SF-6). For all tested concretes, we analyzed the kinetics of the transformation of a given cementitious material from a viscous liquid to a pseudo-solid body. The studies of the kinetics of phase transformation processes allowed us to observe a certain characteristic tendency. Namely, at the beginning of the process, a fast increase in the analyzed parameter was observed, and then its declining increase. A graphical interpretation of such trends is presented by a logistic curve. The logistic trend in the class of non-linear models of development tendencies is of particular importance in this case due to the conditions of the analyzed transformations of cementitious materials from viscous bodies to pseudo-solid bodies. It is the logistic trend where the mathematical form is represented by a logistic curve characterizing the rise in population size under the conditions of a limited potential of the environment. Population is understood here as the increasing mass of the solid phase of the hardening cementitious material (CS (t)), and the limited potential of the environment is understood here as a finite, constantly decreasing mass of viscous liquid (CL (t)). We can also observe a high level of r^2^ determination in all approximations of the measurement results with the logistic curve. The lowest index was observed for concrete SP-2, and it was 0.984.

Based on Figure 10 and Figure 11, we can observe a rise in the solid phase during concrete hardening and a loss of viscous liquid during this time. The mentioned rise is particularly evident after 7, 14 and 28 days. Then, after one year and during the study up to 5 years, these values stabilize, and the fluctuations observed in Figure 3, Figure 4, Figure 5, Figure 6, Figure 7, Figure 8 and Figure 9 result from the standard error presented in the description of the parameters of the logistic models and from the uncertainty of measurements.

Critical times for all samples were determined. The correlation coefficient is at the level of 0.99, meaning that in each case described in this paper, the logistic function describes at least 98% of the results of the experiment.

## Figures and Tables

**Table 1:** Components and physical parameters of concrete mixtures.

Parameters	Type of Concrete Mixture						
	PC	SP-1	SP-2	SP-3	SF-4	SF-5	SF-6
W/(C + SF)	0.52	0.52	0.47	0.42	0.42	0.37	0.32
C (kg/m^3^)	340	345	363	394	320	348	388
SF (kg/m^3^)	-	-	--		36	39	43
SP (kg/m^3^)	-	4.310	4.540	4.925	8.900	9.675	10.781
P (kg/m^3^)	989	982	988	985	1003	992	988
G (kg/m^3^)	989	982	988	985	1003	992	988
W (kg/m^3^)	177	177	168	163	144	137	132
ρ_B_ (kg/m^3^)	2495	2490	2512	2532	2515	2518	2550
ρ_SB_ (kg/m^3^)	2519	2514	2533	2545	2552	2564	2577
s (-)	0.990	0.990	0.992	0.995	0.985	0.982	0.990
j (-)	0.001	0.001	0.008	0.005	0.015	0.018	0.010
V_a_ (dm^3^/m^3^)	10	10	8	5	15	18	10
V_e_-B_e_ (s)	10.5	7.0	8.0	8.0	9.5	10.5	9.0
f_c, cube_ (MPa) after 28 days in hydroisolated condition (18 ± °C)	50.4	53.5	63.7	77.8	77.7	86.4	93.5

The table contains: W/(C + SF)—water binder ratio; C, SF, SP (40% water solution of superplasticizer), P, G and W—content of cement, silica fume, superplasticizer, sand, basalt grit and water in 1 m^3^ of concrete mixture, respectively. It also contains: ρ_B_, ρ_SB_, s, j, V_a_, V_e_-B_e_ and f_c,cube_—apparent density and density of concrete mixture, tightness and cavity, volume of air pores, consistency of concrete mixture and compression strength of concrete, respectively. SP contains the remaining water in the formula W/(C + SF).

**Table 2:** Initial parameters of cementitious materials.

Parameters	Type of Concrete Mixture						
	PC	SP-1	SP-2	SP-3	SF-4	SF-5	SF-6
*C_L_*_,0_	1506	1508.31	1523.54	1546.93	1511.9	1525.68	1561.78
*C_s_*_,0_	989	982	988	985	1003	992	988
ρ_B_	2495	2490	2515	2532	2515	2518	2550

**Table 3:** Maximum values of the parameters of the structures of the analyzed cementitious materials [29].

Parameter	PC	SP-1	SP-2	SP-3	SF-4	SF-5	SF-6
*α*_max_	1.000	1.000	1.000	0.957	1.000	0.937	0.810
*x*_max_	0.644	0.644	0.790	0.937	0.742	0.796	0.872
*R*_max_ (MPa)	78.7	120.8	147.6	175.2	114.2	131.7	160.9
*R*_0_ (MPa)	125.11	187.26	187.26	187.26	251.16	251.16	251.16

**Table 4:** Conversion degree of cementitious materials.

Hardening Time (24 h)	Degree of Conversion α (-)						
	PC	SP-1	SP-2	SP-3	SF-4	SF-5	SF-6
0.001	0	0	0	0	0	0	0
0.5	0.065	0.053	0.061	0.056	0.068	0.087	0.121
1	0.207	0.096	0.119	0.109	0.131	0.147	0.222
3	0.393	0.251	0.255	0.244	0.319	0.382	0.347
7	0.534	0.371	0.330	0.349	0.513	0.563	0.458
14	0.651	0.468	0.467	0.434	0.672	0.711	0.564
28	0.736	0.534	0.508	0.519	0.793	0.776	0.699
365	0.802	0.706	0.671	0.686	0.893	0.926	0.921
730	0.824	0.739	0.705	0.723	0.917	0.944	0.941
1095	0.839	0.757	0.721	0.742	0.929	0.951	0.950
1460	0.846	0.767	0.731	0.754	0.934	0.956	0.956
1825	0.854	0.776	0.743	0.763	0.939	0.959	0.956

**Table 5:** Results of numerical analyses.

Parameters	Concrete Type						
	PC	SP-1	SP-2	SP-3	SF-4	SF-5	SF-6
*C_s_*_,max_	2207.87	2104.02	2072.29	2125.44	2366.94	2388.06	2447.66
*C_s_*_,mx_/2	1103.94	1052.01	1036.14	1062.72	1183.47	1194.03	1223.82
*C_s_*_,max_/(1 + b)	990.69	987.75	994.96	994.32	1095.20	1100.03	1217.78
*t_cr_*	0.742	1.007	0.969	1.203	0.937	0.853	0.117

## Data Availability

Not applicable.
